# Effectiveness of Community-Wide and Individual High-Risk Strategies to Prevent Diabetes: A Modelling Study

**DOI:** 10.1371/journal.pone.0052963

**Published:** 2013-01-04

**Authors:** Douglas G. Manuel, Laura C. Rosella, Meltem Tuna, Carol Bennett, Thérèse A. Stukel

**Affiliations:** 1 The Clinical Epidemiology Program, Ottawa Hospital Research Institute, Ottawa, Ontario, Canada; 2 The Departments of Family Medicine and Epidemiology and Community Medicine, University of Ottawa, Ottawa, Ontario, Canada; 3 The Institute for Clinical Evaluative Sciences, Toronto and Ottawa, Ontario, Canada; 4 Dalla Lana School of Public Health, University of Toronto, Toronto, Ontario, Canada; 5 Public Health Ontario, Toronto, Ontario, Canada; 6 Department of Health Policy, Management and Evaluation, University of Toronto, Toronto, Ontario, Canada; Fundación para la Prevención y el Control de las Enfermedades Crónicas No Transmisibles en América Latina (FunPRECAL), Argentina

## Abstract

**Background:**

Diabetes has been described as one of the most important threats to the health of developed countries. Effective population strategies to prevent diabetes have not been determined but two broad strategies have been proposed: “high-risk” and “community-wide” strategies.

**Methods:**

We modelled the potential effectiveness of two strategies to prevent 10% of new cases of diabetes in Ontario, Canada over a 5-year period. The 5-year risk of developing physician-diagnosed diabetes was estimated for respondents to the Canadian Community Health Survey 2003 (CCHS 2.1, N = 26 232) using a validated and calibrated diabetes risk tool (Diabetes Population Risk Tool [DPoRT]). We estimated how many cases of diabetes could be prevented using two different strategies: a) a community-wide strategy that would uniformly reduce body mass index (BMI) in the entire population; and b) a high baseline risk strategy using either pharmacotherapy or lifestyle counselling to treat people who have an increased risk of developing diabetes.

**Results:**

In 2003, the 5-year risk of developing diabetes was 4.7% (383 600 new diagnosed cases of diabetes in 8 189 000 Ontarians aged 20+) and risk was moderately diffused (0.5%, 3.1% and 17.9% risk in the 1^st^, 5^th^ (median) and 10^th^ deciles of risk). A 10% reduction in new cases of diabetes would have been achieved under any of the following scenarios: if BMI was 3.5% lower in the entire population; if lifestyle counselling covered 32.2% of high-risk people (371 900 of 1 155 000 people with 5 year diabetes risk greater than 10%); or, if pharmacotherapy covered 65.2% of high-risk people.

**Conclusions:**

Prevention using pharmacotherapy alone requires unrealistically high coverage levels to achieve modest population reduction in new diabetes cases. On the other hand, in recent years few jurisdictions have been able to achieve a reduction in BMI at the population level, let alone a reduction of BMI of 3.5%.

## Introduction

Diabetes Mellitus (DM) has been described as one of the most important threats to the health of people in developed countries [1]. The dramatic rise in diabetes prevalence has been related to a corresponding increase in obesity – the main risk for developing diabetes.

Effective population strategies to prevent diabetes have not been determined but two broad strategies have been proposed for type 2 diabetes: “high-risk” and “community-wide” strategies [2]. The high-risk strategy identifies individual people at high risk of developing diabetes and offers them preventive therapy. Two preventive interventions—pharmacotherapy, such as metformin, and lifestyle counselling for diet and exercise—have been shown to be efficacious in clinical trials, but the level of effectiveness for preventing diabetes in entire populations is not known [3]. The community-wide strategy follows the approach of Rose, who proposed that interventions with a small individual benefit can have a large collective effect when they target an entire population, particularly when risk is diffused throughout the population [4], [5]. Advocates of this approach argue that the dramatic increase in diabetes is a consequence of an obesogenic society and that reducing diabetes is only possible by correcting the root causes of obesity—such as a sedentary lifestyle and the wide availability of inexpensive energy-dense food [6]–[8]. Critics point to the increasing level of obesity in most countries despite widespread concerns, and to a lack of population-based intervention studies.

In this study we compared the number of diabetes cases that could be prevented or postponed with two modelled strategies:

Individual prevention (or high baseline risk) strategy – treating individuals who are at high risk of developing diabetes with preventive interventions. We examined the preventive benefit of two different interventions: pharmacotherapy and lifestyle counselling.

Community-wide strategy – lowering weight (body mass index [BMI]) uniformly in the entire population.

**Figure 1:**
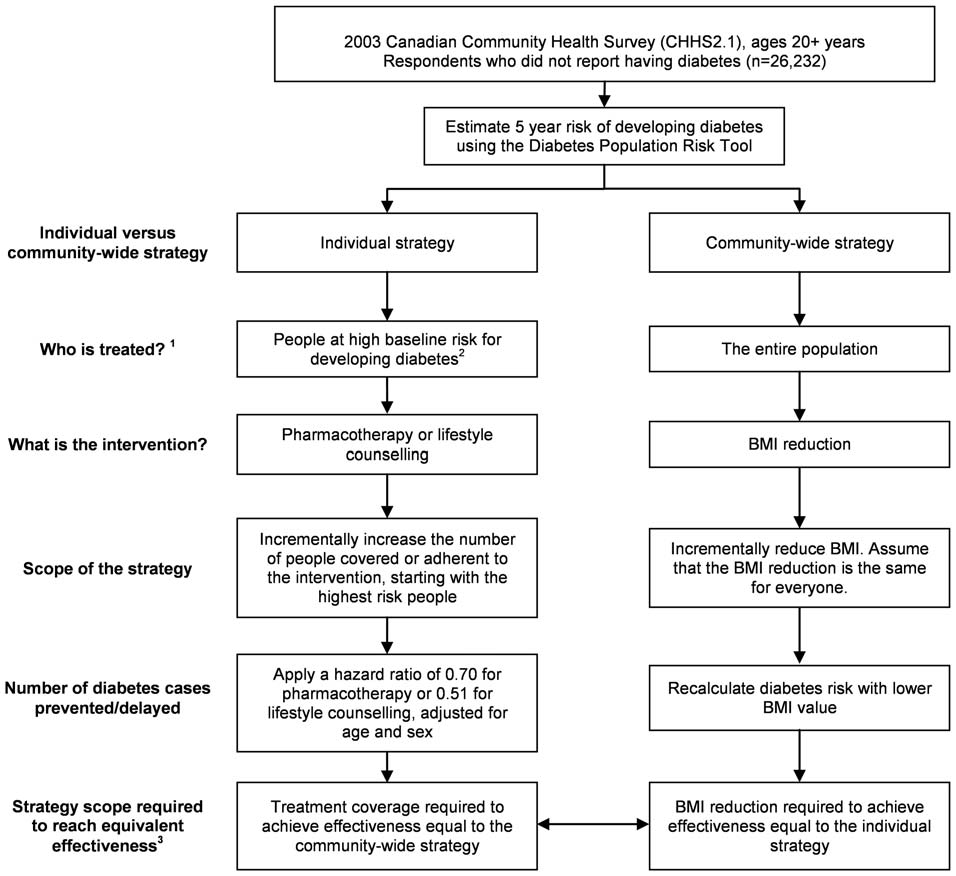
Process to determine number of people treated and diabetes cases prevented under each prevention strategy. Legend: ^1^ In sensitivity testing, only people with an elevated BMI (BMI>25) were treated. ^2^ The initial high-risk strategy scenario targeted people with a 5 year diabetes risk greater than 10%. An alternative approach targeted the highest risk people, followed by people at incremental lower risk. ^3^ The primary scenario was the strategy scope required to prevent diabetes by 10% between 2003 and 2008.

For both strategies, we first calculated the population risk of diabetes (see Figure 1) in a sample of Ontarians using a validated prediction tool (DPoRT) [9]. Next, we calculated the population benefit of different preventive scenarios. We compared the scope of the intervention required for each of the two strategies to have been equally effective in preventing diabetes over a 5-year period (2003–2008) in Ontario, Canada. For the community-wide strategy, we defined scope as incremental reductions of weight in the entire population, which result in a corresponding BMI reduction. For the individual prevention strategy, we examined incrementally larger numbers of people covered by or adherent to either pharmacotherapy or lifestyle counselling. The primary scenario was the strategy scope required to have prevented 10% of new diabetes cases. As an additional measure of effectiveness, we calculated the number needed to treat for the individual strategy.

## Methods

### Ethics Statement

The study was approved by the research ethics board of Sunnybrook Health Sciences Centre.

### Data sources used to estimate risk factors for diabetes

We used data from the Ontario portion of the 2003 Canadian Community Health Survey (CCHS 2.1). We included all respondents over age 20 who did not report they had diabetes (n = 26 232, weighted population = 8 189 000). The Canadian Community Health Survey is an ongoing survey designed to provide cross-sectional estimates of health determinants, health status and health system use at a sub-provincial level.[Beland, 2005] The survey base is the non-institutional household population aged 12 or older in all provinces and territories, except members of the regular Canadian Forces and residents of Indian reserves, Canadian Forces bases (military and civilian), and some remote areas. It is representative of 98% of the population. Each survey respondent has a survey weight that reflects the probability of being selected from the study base. Meaning, the sum of the survey weight for all respondents corresponds to the population count for the study base. In this way, survey estimates that are weighted reflect the total count at the population level.

### Estimating diabetes risk using the Diabetes Population Risk Tool

Each respondent's 5-year risk of developing diabetes was estimated using the Diabetes Population Risk Tool (DPoRT). DPoRT was originally developed using the 1996 National Population Health Survey – routinely-collected, self-reported data on health behaviours and sociodemographic characteristics – to predict the risk of developing physician-diagnosed diabetes. DPoRT was based on sex-specific Weibull survival models for persons >20 years, free of DM and not pregnant. Predictive variables include: age, sex, body mass index (BMI), ethnicity, immigrant status (for women), education, smoking status, history of hypertension and heart disease .(See Table S1 for algorithm formula and Table S2 for an example of its use for risk calculation). All variables that were used to derive DPoRT were also available in the study data.

In the original development and validation data sources, DPoRT had predictive accuracy/calibration – defined as how well the predictive probability of disease closely agrees with the observed outcome – that is similar or better than most other diabetes risk algorithms that have been developed using clinical measures such as measured waist circumference or diabetes-specific questions such as family history of diabetes [10], [11]. DPoRT's risk discrimination – the ability to differentiate between those who are high risk and those who are at low risk– is modestly less or comparable to other risk algorithms.

**Figure 2:**
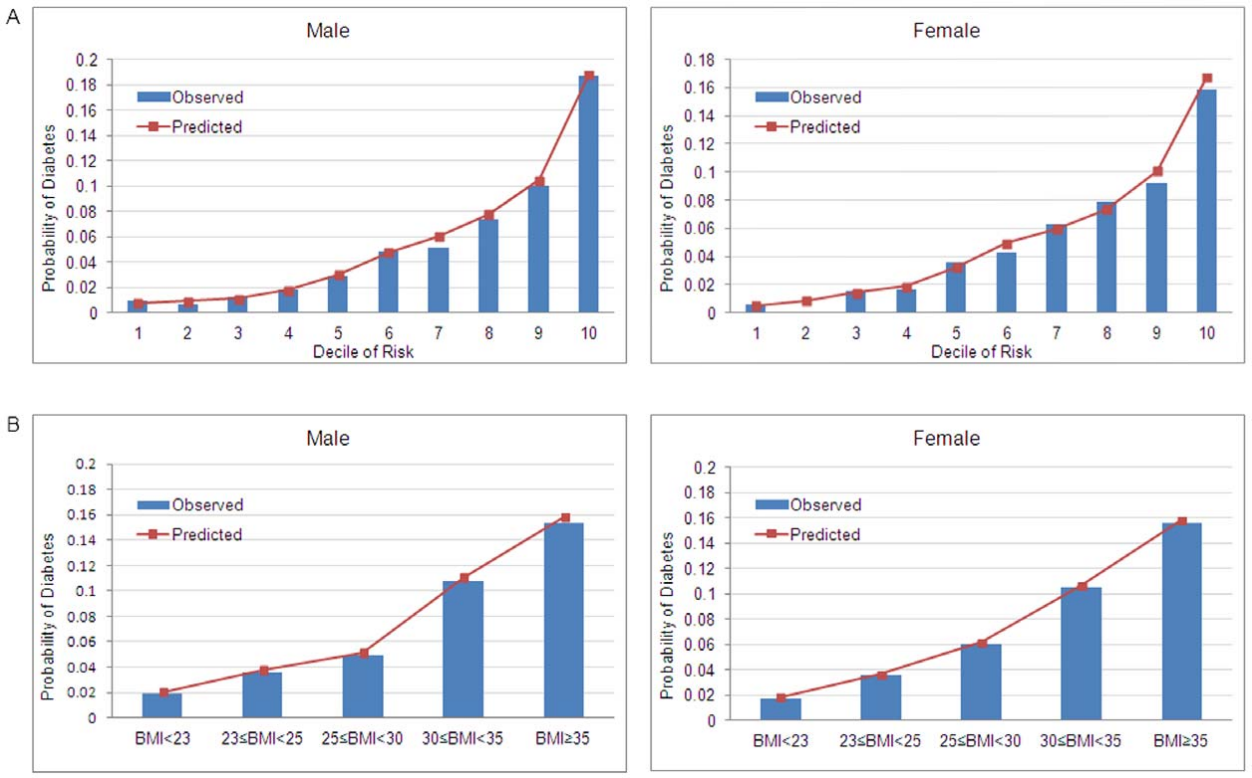
Five-year risk of diabetes by decile of risk and body mass index, Ontario, 2003. Legend: The predicted five-year probability of diabetes (DM) calculated using the Diabetes Population Risk Tool (DPoRT) compared to the observed probability of diabetes, by (A) deciles of risk and (B) body mass index categories. The observed probability of diabetes was ascertained using the Ontario Diabetes Database individually linked to the Ontario sample of the Canadian Community Health Survey.

We validated DPoRT for the CCHS 2.1 to assess predictive accuracy. Validation focused on whether DPoRT accurately predicted risk of diabetes for the study population – meaning whether the number of predicted cases of diabetes closely approximated the number of cases of diabetes that actually occurred in our study population over a 5-year follow-up period. Validation was performed by individually linking the CCHS 2.1 cohort to a population-based registry of physician-diagnosed diabetes. The risk algorithm maintained discrimination (C-statistic = 0.76) and predictive accuracy (χ2H–L = 4.4 males and 6.9 females) after recalibration (observed versus predicted 5-year diabetes risk = 4.6% versus 4.7%, Figure 2a). Discrimination and calibration were also maintained across BMI scores (see Figure 2b) and other subgroups.

### Comparison of population intervention strategies

Figure 1 shows how the population intervention effectiveness of the two strategies was compared. For the community-wide strategy, the target population was all Ontarians age 20 years and older without diabetes in 2003. For the individual strategy, the target population was defined as people with a 5-year risk of diabetes greater than 10%. As an alternative approach, the individual strategy targeted people at highest risk and then incrementally increased strategy scope to include people with lower diabetes risk.

Next, we calculated intervention effectiveness for each strategy assuming different levels of strategy scope. Effectiveness was defined as the number of diabetes cases prevented in the next 5 years. For the community-wide strategy, effectiveness was calculated for different levels of BMI reduction by lowering each respondent's weight, re-calculating BMI, and then using DPoRT to re-estimate the number of people who would develop diabetes in 5 years. The preventive benefit of weight reduction was the difference between the number of diabetes cases calculated using respondents' original reported weights and then re-calculated using the lower, adjusted weight.

For the individual strategy, preventive benefit was calculated using two different approaches. The first approach answered the following question: what intervention coverage among people with diabetes risk greater than 10% would be required to prevent diabetes by 2.5%, 10% and 20%? The second approach answered the same question but for incrementally larger coverage of the overall population, starting with the highest-risk people. We calculated population intervention effectiveness (i.e., the percent of new diabetes cases prevented) using the product of the 5-year baseline risk and an estimate of relative efficacy for each intervention:
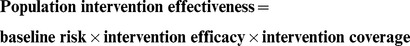
Where the baseline risk of disease is the risk or probability of developing disease calculated using DPoRT, the intervention efficacy was estimated from a meta-analysis on diabetes interventions conducted by Gillies et al. (pharmacological treatment hazard ratio [HR] = 0.7 and lifestyle counselling HR = 0.51) [3], and intervention coverage is the percent of the target population that is identified, offered and adherent to the preventive intervention.

In sensitivity analyses, we restricted the target population for both strategies to people whose BMI was greater than 25, because pharmacological and lifestyle treatment may not be efficacious in people with normal weight (and are not indicated for this group) and there is concern that a community-wide strategy for weight reduction may be harmful when targeted to people with normal weights [12].

## Results

The 5-year predictive risk of diabetes in Ontario study population in 2003 was 4.7% (383 600 new cases using the study weights). The median BMI was 26.1 (IQR 4.9) for males and 24.6 (6.3) for women. The median age for men and women was 47 (27) and 51 (29) years respectively. (See Table S3 for additional characteristics of the Ontario population.)

Figures 2a and b show that population risk for diabetes in Ontario was moderately diffused. Women who were severely obese (BMI>35) were at a markedly increased risk of diabetes, but fourfold more cases of diabetes occurred in women who were overweight and obese (BMI 25 to 35) (not shown). Men in the top decile of diabetes risk had over 34 times the risk of people in the lowest decile, but only the top two deciles of men and women had a 5-year diabetes risk greater than 10%.

Table 1 (also see Table S4) shows the coverage levels for pharmacotherapy or lifestyle counselling (individually targeted strategies) or BMI reduction (community-wide strategy) that were required to achieve equivalent diabetes prevention. For the community-wide strategy, a 10% reduction in new diabetes cases (38 500 cases prevented) was achieved with a 3.5% decrease in mean population weight. For the individual strategy, a 10% reduction in new diabetes cases was achieved when lifestyle counselling covered 32% (n = 371 900) of high-risk people (defined as having a 5-year diabetes risk of 10% or greater) or 4.5% of the total Ontario adult population with the highest diabetes risk (5-year diabetes risk ≥15.8%). The estimate of 4.5% of the adult population represents the most efficient lifestyle intervention strategy possible to achieve a 10% reduction in new cases of diabetes – the strategy where people with the highest risk of diabetes (or greatest benefit from therapy) are all targeted and adherent to therapy. Pharmacotherapy required 65% coverage (n = 752 800) of high-risk people or 9.2% of the total population, corresponding to all Ontarians whose diabetes risk was ≥11.2%.

**Table 1:** Strategy scope required to prevent diabetes by 2.5%, 10% or 20%.

Intervention Strategy	Target level of diabetes prevention (number of cases prevented)		
	2.5% (9600)	10% (38 500)	20% (76 700)
High-risk strategy - Pharmacotherapy			
Intervention coverage required:			
High-risk population*	10.2%	65.2%	>100%
Total population	1.4%	9.2%	30.6%
Corresponding to 5-year diabetes risk ≥	20.0%	11.2%	5.9%
Number needed to treat	11.9	19.5	31.1
High-risk strategy - Lifestyle counselling			
Intervention coverage required:			
High-risk population*	5.6%	32.2%	88.0%
Total population	0.8%	4.5%	12.4%
Corresponding to 5-year diabetes risk ≥	26.4%	15.8%	10.3%
Number needed to treat	6.6	9.7	13.2
Community-wide strategy			
BMI reduction for all Ontarians	1%	3.5%	7.8%

* High-risk population—people with 5-year risk greater than 10%.

**Figure 3:**
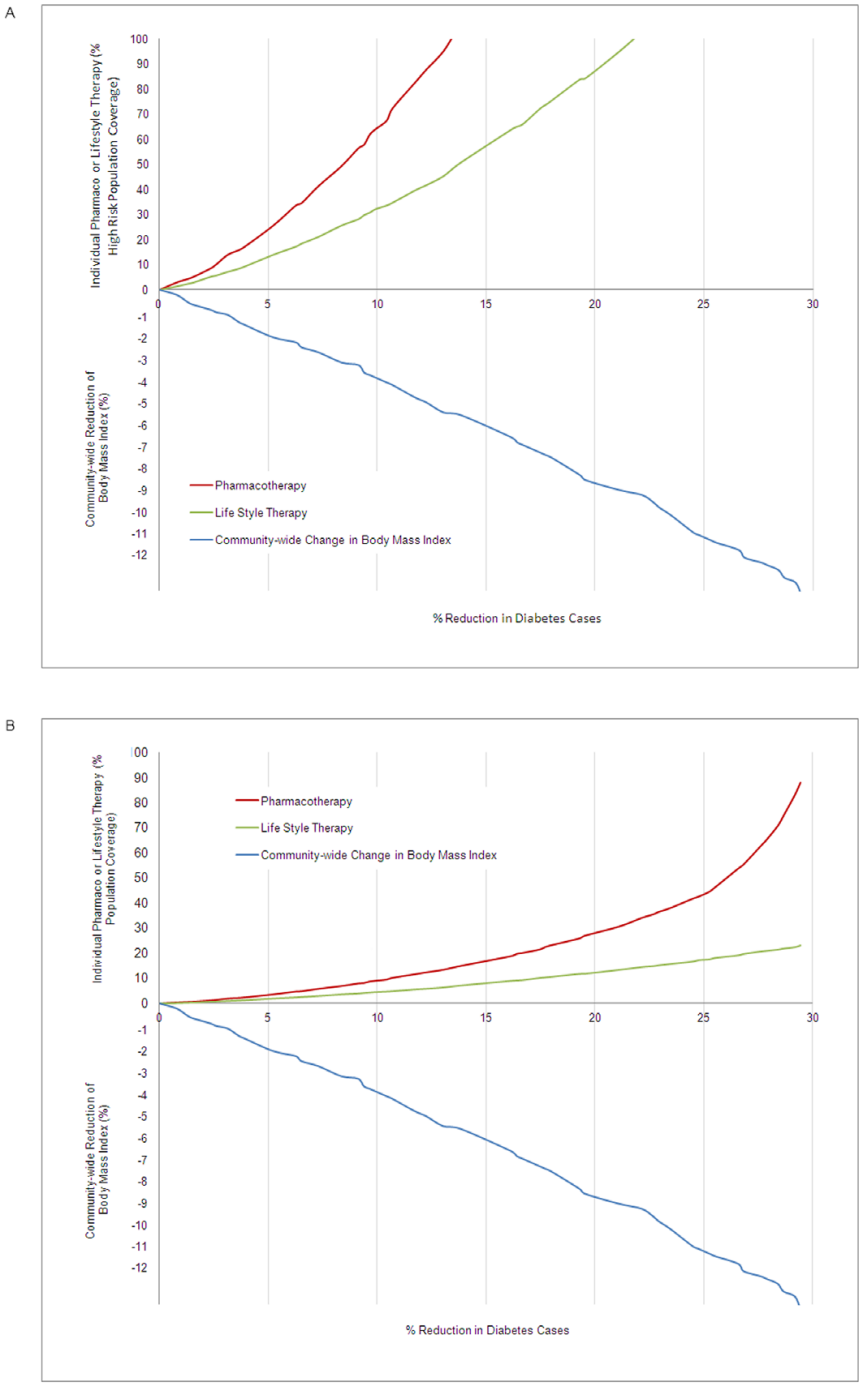
Incremental diabetes prevention based on scope of pharmacotherapy, lifestyle therapy, or community-wide strategy. Legend: High-risk (A) and general (B) population coverage of pharmacotherapy or lifestyle therapy with diabetes preventive benefit equivalent to different levels of body mass index (BMI) reduction. Greater reductions of diabetes (x-axis) require either greater reductions of indivdual therapy or community-wide BMI reduction. For example, for people at high-risk of developing diabetes (A), a 10% reduction in new cases of diabetes would require indivdual therapy coverage of 65% for pharmacotherapy or 32% for lifestyle therapy. Alternatively, a community-wide 3.5% BMI reduction would achieve the same 10% reduction in diabetes.

Table 1 also shows the strategy scope required to prevent or delay 2.5 or 20% of diabetes cases. Figure 3 (and Figure S1) shows the same data but also illustrates incremental reductions in the number of diabetes cases. Both strategies require a proportionately larger scope to achieve higher preventive effectiveness, but more so for the individual strategy because interventions (lifestyle modification and pharmacotherapy) targeted people at incrementally lower risk. Because people with a normal weight (BMI≤25) contributed to a small amount of population risk, restricting the target population to Ontarians with a BMI>25 resulted in only a modest reduction in predicted effectiveness of both strategies (see Table S4).

## Discussion

Our study suggests that jurisdictions similar to Ontario (industrialized countries with a moderate and growing diabetes prevalence) face a large challenge to achieve even modest diabetes coverage using either individual or community-wide interventions.

Clearly, individual pharmacotherapy, by itself, cannot achieve a 10% reduction in new diabetes cases. Such a reduction would require unrealistically high coverage rates of pharmacotherapy (65% of high-risk people). In the case of hypertension and dyslipidemia, the highest performing countries have only recently achieved coverage rates of 50%, despite the fact that screening and drug therapy have been recommended for several decades and these drugs are the most frequently dispensed in many drug plans [13].

Although reducing weight in the population is undoubtedly feasible, BMI levels in most developed countries are still increasing. A 1% decrease in BMI (the improvement needed to reduce diabetes by 2.5% over 5 years) mirrors the increase in BMI that has been observed in Ontario and other jurisdictions in recent years [14], [15]. Widely ranging approaches to reduce BMI are emerging, but demonstration of their effectiveness is incomplete [8], [15].

Given that diabetes risk is moderately diffused in the population, policy actors should consider an approach that combines both community-wide and individual strategies. Rose showed that when risk is diffused throughout the population—e.g., when there is a wide range in risk of diabetes— a community-wide preventive strategy has greater potential effectiveness compared to a strategy that targets high-risk individuals [16]. Using the same argument, if population risk is concentrated in a small group of high-risk people then an individual strategy is warranted.

Economic analyses comparing community-wide to high-risk interventions is challenging [17], [18]. For individual interventions, cost-effective analyses are reasonable and relatively straightforward. Both pharmacotherapy and lifestyle counselling have been shown to be cost-effective in high-risk populations, with lifestyle counselling being more cost-effective largely because it is a short-term intervention with potentially long-lasting benefit [19], [20]. However, cost-effectiveness will become less favourable as more people at moderate and low risk are treated [19], [20]. Figure 3b graphically presents how the cost-effectiveness will be reduced with a progressively smaller absolute benefit when incrementally larger numbers of people are offered therapy.

Estimating the cost effectiveness of community-wide weight reduction strategies is more challenging with no clearly established methods beyond the evaluation of specific interventions [17]. Furthermore, cost effectiveness estimates for community-wide interventions are not translatable across jurisdictions [21], [22]. The case of smoking reduction, where there have been few cost-effectiveness studies of community-wide interventions such as smoking by-laws, is testimony to the ability to widely implement behavioural change interventions in the absence of economic analyses.

That stated, community-wide strategies that target the obesogenic environment may either be very affordable or very costly, depending on the type of intervention and the way that costs are allocated. Public policy that aims to improve population weights by targeting large populations can be inexpensive to develop and implement but target large numbers of people. For this reason, public policy interventions are often the most cost-effective interventions. However, these approaches are significantly more complicated having a number of legislative, jurisdictional, ethical, and political issues. Indeed, such obesity interventions can be cost-saving, such as unhealthy food and beverage tax and front-of-pack traffic light nutrition labelling [8], [23]. Furthermore, advocates rightly point out that community-wide interventions to address obesity will have benefit for diseases and health outcomes well beyond diabetes [24]. On the other hand, interventions can be very costly. There is much recent discussion about the role of built environment and obesity. Creating communities that enable healthy behaviour requires massive investment in physical infrastructure. In Ontario, the availability of accessible public transportation systems has been identified as a prerequisite in rebuilding neighbourhoods to support high levels of physical activity and low diabetes prevalence. In Toronto alone, over $20 billion (CDN) has been pledged to improve public transportation.

If this study were duplicated in other developed countries, we expect the results would be similar though exact findings would vary depending on the overall level and diffusion patterns of diabetes risk. Compared to a community-wide strategy, an individual strategy will be more effective in countries where population risk is concentrated in the high-risk group. Simply examining population weights can provide insight into the overall level of diabetes risk, and countries with higher weights can expect greater preventive benefit of both strategies. However, in our experience it is difficult to gauge the risk diffusion pattern without explicitly calculating diabetes risk. If a population's weight patterns differ from Ontario's in 2003, then diabetes risk should be estimated to examine the preventive benefit of different community-wide or high-risk strategies.

A limitation of our study was the lack of data on impaired glucose tolerance (IGT) to identify people at high risk of developing diabetes. Most individual intervention studies have targeted people with IGT, whereas we used a multivariate risk tool to define risk. However, the baseline risk assessment using DPoRT had at least two advantages beyond serving as a proxy for IGT. First, our approach of targeting people at high risk is the common practice for other conditions such as heart disease and is becoming common for diabetes. For example, Finland uses a multivariate prediction tool (the FINRISK tool) to stratify participants into different interventions depending on their level of baseline risk for developing diabetes [25]. Other countries are following Finland's lead. Second, we purposefully sought to assess a high baseline risk approach as an individual prevention strategy. Generally, targeting people assessed by a multi-attribute risk tool such as FINRISK or DPoRT will be more effective and efficient than targeting people with a single raised risk factor, such as IGT [5]. As well, Gillies et al. showed that intervention benefit for both pharmacotherapy and lifestyle counselling was the same over a wide range of baseline risk [3]. If IGT had been available, it would not have changed our assessment of who would benefit from preventive interventions or the estimate of benefit.

Both a strength and limitation of our study is the use of a population health survey and the DPoRT risk tool to estimate baseline risk. Assessing the diffusion of population risk is a cornerstone of population health planning [5], [16], [24], [26], [27]. Currently, multivariate risk tools such as DPoRT are the most accurate approach to assessing baseline risk. DPoRT is particularly attractive because it is accurate (very good discrimination and predictive accuracy/calibration) and requires only information that is captured in large, population health surveys. However, other clinical risk prediction tools for diabetes such as FINRISK are even more discriminating because they include questions about IGT and other clinical specifications. If we had been able to use such clinical risk tools (not usually possible because they require specific clinical data not routinely captured in population surveys), we may have found that population risk is more concentrated in high-risk people, which would increase the apparent effectiveness and efficiency of an individual (high baseline risk) strategy.

## Conclusions

Targeting individuals at high baseline risk should not be the sole approach to diabetes prevention in populations with moderate risk diffusion. Unrealistically large proportions of the total population would have to be adherent to therapy in order for individual strategies to achieve important levels of diabetes prevention. However, there are equally large challenges for achieving modest weight reductions in the population that would result in meaningful changes to diabetes risk, given that weights continue to increase in most industrialized countries and important interventions – such as changing built environments – are potentially very complex and difficult to implement. Where risk for diabetes is moderately diffused throughout the population—as was the case in Ontario, Canada, in 2003 and is also the likely situation in most industrial populations— both individual and community-wide strategies should be both considered, given that diabetes risk is moderately diffused in the population.

## Supporting Information

Figure S1 **High-risk population^1^ coverage of pharma or lifestyle therapy with a preventive benefit equivalent to different levels of BMI reduction.** Legend: ^1^High-risk population —people with a 5-year risk of diabetes (DM) greater than 10%. The two lines represent diabetes prevention that is achieved with equal scope of either individual interventions (diabetes prevention or delay using pharmacotherapy or lifestyle therapy) compared to community-wide weight reduction of body mass index (BMI). For example, a 10% reduction in new diabetes cases could be achieved with a 3.5% reduction in community-wide BMI or 65% coverage of individual prevention using pharmacotherapy.
(TIF)

Table S1 Calibrated Diabetes Population Risk Tool (DPoRT) functions for predicting 5-year risk of physician diagnosed diabetes for females and males.
(DOC)

Table S2 Calculating 5-year diabetes risk using the Diabetes Population Risk Tool (DPoRT) - two hypothetical profiles.
(DOC)

Table S3 Characteristics of the study population (Ontario respondents, age 20+, from the 2003 Canadian Community Health Survey).
(DOC)

Table S4 Examples of population characteristics, diabetes outcomes and strategy effectiveness for different target populations and strategy scope.
(DOC)
