# Supplementary figures and images for: Effectiveness of Community-Wide and Individual High-Risk Strategies to Prevent Diabetes: A Modelling Study
Source: PLoS One. 2013 Jan 4;8(1):e52963. doi: 10.1371/journal.pone.0052963 (PMC3537737; doi:10.1371/journal.pone.0052963)

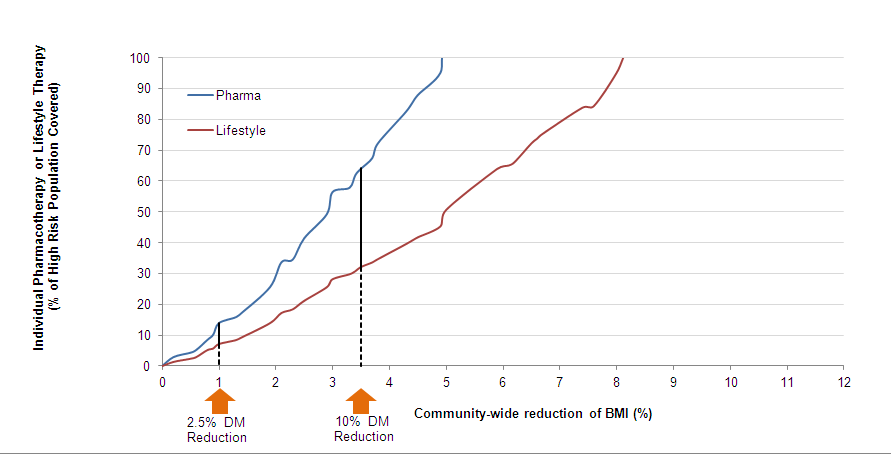

Supplement: Figure S1 — High-risk population1 coverage of pharma or lifestyle therapy with a preventive benefit equivalent to different levels of BMI reduction. Legend: 1High-risk population —people with a 5-year risk of diabetes (DM) greater than 10%. The two lines represent diabetes prevention that is achieved with equal scope of either individual interventions (diabetes prevention or delay using pharmacotherapy or lifestyle therapy) compared to community-wide weight reduction of body mass index (BMI). For example, a 10% reduction in new diabetes cases could be achieved with a 3.5% reduction in community-wide BMI or 65% coverage of individual prevention using pharmacotherapy. (TIF) [file pone.0052963.s001.tif]
