# Supplementary material for: Effectiveness of Community-Wide and Individual High-Risk Strategies to Prevent Diabetes: A Modelling Study
Source: PLoS One. 2013 Jan 4;8(1):e52963. doi: 10.1371/journal.pone.0052963 (PMC3537737; doi:10.1371/journal.pone.0052963)
Supplement: Table S1 — Calibrated Diabetes Population Risk Tool (DPoRT) functions for predicting 5-year risk of physician diagnosed diabetes for females and males. (DOC) [file pone.0052963.s002.doc]

Table S1. Calibrated Diabetes Population Risk Tool (DPoRT) functions for predicting 5-year risk of physician diagnosed diabetes for females and males.

| Females. | |  |  | Males | | |  |
| --- | --- | --- | --- | --- | --- | --- | --- |
| Risk Factor | Value |  |  | Risk Factor | | Value | |
| Intercept | 10.75 |  |  | Intercept | 10.46 | |  |
| Hypertension | -0.53 |  |  | Hypertension | -0.53 | |  |
| Ethnicity | -0.39 |  |  | Ethnicity | -0.47 | |  |
| Immigrant | -0.16 |  |  | Heart Disease | -0.21 | |  |
| Education | 0.14 |  |  | Smoking | 0.02 | |  |
| Age <45 BMI 23-25 | -1.79 |  |  | Education | 0.15 | |  |
| Age <45 BMI 25-30 | -1.6 |  |  | Age <45 BMI 23-25 | 0.05 | |  |
| Age <45 BMI 30-35 | -2.88 |  |  | Age <45 BMI 25-30 | -0.15 | |  |
| Age <45 BMI 35+ | -2.89 |  |  | Age <45 BMI 30-35 | -1.27 | |  |
| Age <45 BMI Unknown | -0.99 |  |  | Age <45 BMI 35+ | -1.69 | |  |
| Age 45-65 and BMI<23 | -1.57 |  |  | Age 45+ BMI <23 | -0.58 | |  |
| Age 45-65 and BMI 23-25 | -1.98 |  |  | Age 45+ BMI 23-25 | -1.19 | |  |
| Age 45-65 BMI 25-30 | -2.61 |  |  | Age 45+ BMI 25-30 | -1.46 | |  |
| Age 45-65 BMI 30-35 | -3.1 |  |  | Age 45+ BMI 30-35 | -2 | |  |
| Age 45-65 BMI 35+ | -3.63 |  |  | Age 45+ BMI 35+ | -2.4 | |  |
| Age 45-65 BMI Unknown | -3.26 |  |  | Scale | 0.85 | |  |
| Age 65+ and BMI <23 | -2.26 |  |  |  | | |  |
| Age 65+ and BMI 23-25 | -2.61 |  |  |  |  | |  |
| Age 65+ BMI 25-30 | -2.57 |  |  |  |  | |  |
| Age 65+ BMI 30-35 | -2.98 |  |  |  |  | |  |
| Age 65+ BMI 35+ | -3.04 |  |  |  |  | |  |
| Age 65+ BMI Unknown | -3.53 |  |  |  |  | |  |
| Scale | 0.87 |  |  |  |  | |  |
| Abbreviations: BMI = Body mass index (kg/m^2^) | | | | | | |  |
