# Supplementary material for: Effectiveness of Community-Wide and Individual High-Risk Strategies to Prevent Diabetes: A Modelling Study
Source: PLoS One. 2013 Jan 4;8(1):e52963. doi: 10.1371/journal.pone.0052963 (PMC3537737; doi:10.1371/journal.pone.0052963)
Supplement: Table S2 — Calculating 5-year diabetes risk using the Diabetes Population Risk Tool (DPoRT) - two hypothetical profiles. (DOC) [file pone.0052963.s003.doc]

Table S2. Calculating 5-year diabetes risk using the Diabetes Population Risk Tool (DPoRT) - two hypothetical profiles.

| **Profile 1:** Female, 30 years old, height=1.70 m, weight=69 kg (BMI=24 kg/m^2^), no hypertension, white, not immigrant, has post secondary education. **Five year risk of being diagnosed with diabetes using the DPoRT is 2.3%.**  **Profile 2:** Male, 50 years old, height=1.85 m, weight=129 kg (BMI=38 kg/m^2^), hypertensive, white, has heart disease, smoker, has less then secondary school education. **Five year risk of being diagnosed with diabetes using the DPoRT is 32.2%.** |
| --- |

| **µ** = 10.75-0.53*(Hypertension(no) - 0.15) - 0.39*(Ethnicity(no) - 0.18)  - 0.16*(Immigrant(no) - 0.31) + 0.14*(Education(Post secondary) - 0.62)  - 1.79*(Age <45 BMI 23-25 (yes) - 0.09) - 1.6*(Age <45 BMI 25-30 (no) - 0.1)  - 2.88*(Age <45 BMI 30-35 (no) - 0.04) - 2.89*(Age <45 BMI 35+(no) - 0.02)  - 0.99*(Age <45 BMI Unknown(no) - 0.02) - 1.57*(Age 45-65 and BMI<23(no) - 0.09)  - 1.98*(Age 45-65 and BMI 23-25 (no) - 0.06) - 2.61*(Age 45-65 BMI 25-30 (no) - 0.1)  - 3.1*(Age 45-65 BMI 30-35 (no) - 0.04) - 3.63*(Age 45-65 BMI 35+(no) - 0.02)  - 3.26*(Age 45-65 BMI Unknown(no) - 0.02) - 2.26*(Age 65+ and BMI 23-24(no) - 0.05)  - 2.61*(Age 65+ and BMI<23 (no) - 0.03) - 2.57*(Age 65+ BMI 25-30 (no) - 0.05)  - 2.98*(Age 65+ BMI 30-35(no) - 0.02) - 3.04*(Age 65+ BMI 35+(no) – 0.05)  - 3.53*(Age 65+ BMI Unknown(no) - 0.01)  **µ** = 10.75-0.53*(0 - 0.15) - 0.39*(0 - 0.18) - 0.16*(0 - 0.31) + 0.14*(0 - 0.62)  - 1.79*(1 - 0.09) - 1.6*(0 - 0.1) - 2.88*(0 - 0.04) - 2.89*(0 - 0.02) - 0.99*(0 - 0.02) - 1.57*(0 - 0.09)  - 1.98*(0 - 0.06) - 2.61*(0 - 0.1) - 3.1*(0 - 0.04) - 3.63*(0 - 0.02) - 3.26*(0 - 0.02) - 2.26*(0 - 0.05)  - 2.61*(0 - 0.03) - 2.57*(0 - 0.05) - 2.98*(0 - 0.02) - 3.04*(0 – 0.05) - 3.53*(0 - 0.01)  **µ** =10.77  m=(log(365.25*5) - **µ** ) / σ = (7.51 – 10.77) / 0.87 = -3.75  **5-year predicted risk for developing diabetes:**  P= 1-exp(-e ^-3.75^)  P= 0.023 or 2.3% |
| --- |
