# Supplementary material for: Effectiveness of Community-Wide and Individual High-Risk Strategies to Prevent Diabetes: A Modelling Study
Source: PLoS One. 2013 Jan 4;8(1):e52963. doi: 10.1371/journal.pone.0052963 (PMC3537737; doi:10.1371/journal.pone.0052963)
Supplement: Table S3 — Characteristics of the study population (Ontario respondents, age 20+, from the 2003 Canadian Community Health Survey). (DOC) [file pone.0052963.s004.doc]

Table S3. Characteristics of the study population (Ontario respondents, age 20+, from the 2003 Canadian Community Health Survey).

| **Risk Factor** n (%) | **Men**  11 879 (48.3) | **Women**  14 353 ( 54.7) |
| --- | --- | --- |
| Body Mass Index (Kg/m^2^) ^Ŧ^ | 26.1 (4.9) | 24.6 (6.3) |
| Age ^Ŧ^ | 47 (27) | 51 (29) |
|  |  |  |
| Age <45 | 5332 (44.9) | 5691 (39.6) |
| 45≤Age<65 | 4092 (34.5) | 4856 (33.8) |
| Age≥65 | 2455 (20.7) | 3806 (26.5) |
|  |  |  |
| BMI<23 | 2120 (17.9) | 4871 (33.9) |
| 23≤BMI<25 | 2579 (21.7) | 2596 (18.1) |
| 25≤BMI<30 | 5096 (42.9) | 4198 (29.3) |
| 30≤BMI<35 | 1647 (13.9) | 1544 (10.8) |
| BMI≥35 | 437 (3.7) | 661 (4.61) |
| BMI = missing | 0 | 483 (3.4) |
|  |  |  |
| Non-white | 1221 (10.3) | 1316 (9.2) |
| Immigrant | 2540 (21.4) | 2989 (20.8) |
| Hypertension | 2077 (17.5) | 2958 (20.6) |
| Current Smoker | 3241 (27.3) | 3275 (22.8) |
| Physical Activity - Mets (kcal/day) ^Ŧ^ | 1.7 (2.7) | 1.4 (2.2) |
| Heart Disease | 972 (8.2) | 984 (6.9) |
| Graduated Post Secondary School | 6539 (55.0) | 7273 (50.7) |
| **Diabetes Risk** |  |  |
| Crude 5-year diabetes incidence rate | 4.9 | 4.5 |

^Ŧ^ median (IQR)
