# Supplementary material for: Effectiveness of Community-Wide and Individual High-Risk Strategies to Prevent Diabetes: A Modelling Study
Source: PLoS One. 2013 Jan 4;8(1):e52963. doi: 10.1371/journal.pone.0052963 (PMC3537737; doi:10.1371/journal.pone.0052963)
Supplement: Table S4 — Examples of population characteristics, diabetes outcomes and strategy effectiveness for different target populations and strategy scope. (DOC) [file pone.0052963.s005.doc]

Table S1. Examples of population characteristics, diabetes outcomes and strategy effectiveness for different target populations and strategy scope.

| **Target Population** | All Ontarians | | | Ontarians with BMI >= 25 | | |
| --- | --- | --- | --- | --- | --- | --- |
| **Strategy** | *High baseline risk strategy*  *(Pharmacotherapy or lifestyle counselling)* | | *Community-wide Strategy*  *(weight reduction)* | *High baseline risk Strategy*  *(Pharmacotherapy or lifestyle counselling)* | | *Community-wide Strategy*  *(weight reduction)* |
| **Strategy Scope** | 5-year baseline DM risk ≥ 10% | 5-year baseline DM risk ≥ 5% | *5% BMI reduction in the entire population* | 5-year baseline DM risk ≥ 10% | 5-year baseline DM risk ≥ 5% | *5% BMI reduction in* Ontarians with BMI ≥ 25 |
| **Population characteristics** |  |  |  |  |  |  |
| Number of people | 1 155 000 | 2 792 000 | 8 189 000 | 1 012 000 | 2 284 000 | 344 000 |
| Body Mass Index (Kg/m^2^) (mean/median) | 30.8/30.3 | 29/28 | 25.6/24.9 | 31.3/30.6 | 30.2/28.9 | 29.2/28.1 |
| Age (mean/median) | 60.7/59 | 58/57 | 45.4/43 | 59.9/58 | 56.1/55 | 47.3/46 |
|  |  |  |  |  |  |  |
| Females, % | 45.4 | 47.1 | 51.3 | 41.3 | 44.2 | 41.8 |
| Age <45, % | 3.3 | 11.4 | 53.3 | 3.8 | 14 | 47 |
| 45≤Age<65, % | 58.9 | 55.5 | 32.4 | 61.1 | 59.3 | 37.5 |
| Age≥65, % | 37.8 | 33.1 | 14.6 | 35.2 | 26.7 | 15.5 |
|  |  |  |  |  |  |  |
| BMI<23 | 0.06 | 4.0 | 29.4 | -- | -- | -- |
| 23≤BMI<25 | 6.4 | 10.9 | 20.4 | -- | -- | -- |
| 25≤BMI<30 | 35.7 | 48.0 | 33.7 | 40.7 | 58.7 | 70.2 |
| 30≤BMI<35 | 36.7 | 23.7 | 10.8 | 41.9 | 28.9 | 22.8 |
| BMI≥35 | 15.3 | 10.1 | 3.5 | 17.4 | 12.4 | 7.2 |
| BMI = missing | 5.9 | 3.3 | 2.2 | - | - | - |
|  |  |  |  |  |  |  |
| Non-white, % | 19.5 | 15.9 | 19.2 | 19 | 13.6 | 15 |
| Immigrant, % | 41.6 | 36.7 | 31.9 | 40.1 | 34.5 | 29 |
| Hypertension, % | 63.5 | 36.2 | 14.9 | 62.2 | 31.5 | 20.5 |
| Current Smoker, % | 13.5 | 17 | 24.3 | 13.7 | 17.4 | 22.6 |
| Physical Activity - Mets (kcal/day) | 1.6/1.1 | 1.7/1.2 | 2/1.4 | 1.6/1.1 | 17./1.2 | 1.9/1.4 |
| Heart Disease, % | 15.2 | 11.6 | 5.1 | 13.6 | 9.3 | 5.9 |
| Graduated Post Secondary School, % | 39.7 | 45.2 | 55.7 | 41.5 | 46 | 54 |
| **Diabetes outcomes** |  |  |  |  |  |  |
| Predicted 5-year baseline incident rate (%) | 14.8 | 10.2 | 4.6 | 14.8 | 10.4 | 7 |
| **Strategy effectiveness** |  |  |  |  |  |  |
| Pharmacotherapy |  |  |  |  |  |  |
| Reduction in baseline risk | 0.6% | 1% | - | 0.5% | 0.9% | - |
| Diabetes cases reduced (n) | 51290 | 85300 | - | 44940 | 71160 | - |
| Number needed to treat | 22.5 | 32.7 | - | 22.5 | 32 | - |
| Lifestyle counselling |  |  | - |  |  | - |
| Reduction in baseline risk | 1% | 1.7% | - | 0.9% | 1.4% | - |
| Diabetes cases reduced (n) | 83770 | 139300 | - | 73400 | 116200 | - |
| Number needed to treat | 13.8 | 20 | - | 13.8 | 19.7 | - |
| 5% reduction in BMI |  |  |  |  |  |  |
| Reduction in baseline risk | - | - | 0.7% | - | - | 0.5% |
| Diabetes cases reduced (n) | - | - | 53410 | - | - | 39640 |
